# Unravelling epigenetic mechanisms in *Cerastoderma edule* genome: a comparison of healthy and neoplastic cockles

**DOI:** 10.1007/s00438-024-02148-z

**Published:** 2024-05-25

**Authors:** Alejandro Viña-Feás, Javier Temes-Rodríguez, André Vidal-Capón, Samuel Novas, Jorge Rodríguez-Castro, Ana Pequeño-Valtierra, Juan José Pasantes, Jose M. C. Tubío, Daniel Garcia-Souto

**Affiliations:** 1 Genomes and Disease, Centre for Research in Molecular Medicine and Chronic Diseases (CiMUS), Universidade de Santiago de Compostela, Santiago de Compostela, Spain; 2 Centro de Investigación Mariña, Universidade de Vigo, Vigo, Spain; 3 Instituto de Investigaciones Sanitarias de Santiago de Compostela (IDIS), Santiago de Compostela, Spain; 4 Department of Zoology, Genetics and Physical Anthropology, Universidade de Santiago de Compostela, Santiago de Compostela, Spain; 5 Department of Biological Sciences, School of Environment, Arts and Society, College of Arts, Sciences & Education (CASE), Florida International University, Miami, FL USA

**Keywords:** Bivalve transmissible neoplasia, Epigenetics, Methylation, Cerastoderma edule, Transposons

## Abstract

Cancer is a multifaceted genetic disease characterized by the acquisition of several essential hallmarks. Notably, certain cancers exhibit horizontal transmissibility, observed across mammalian species and diverse bivalves, the latter referred to as hemic neoplasia. Within this complex landscape, epigenetic mechanisms such as histone modifications and cytosine methylation emerge as fundamental contributors to the pathogenesis of these transmissible cancers. Our study delves into the epigenetic landscape of *Cerastoderma edule*, focusing on whole-genome methylation and hydroxymethylation profiles in heathy specimens and transmissible neoplasias by means of Nanopore long-read sequencing. Our results unveiled a global hypomethylation in the neoplastic specimens compared to their healthy counterparts, emphasizing the role of DNA methylation in these tumorigenic processes. Furthermore, we verified that intragenic CpG methylation positively correlated with gene expression, emphasizing its role in modulating transcription in healthy and neoplastic cockles, as also highlighted by some up-methylated oncogenic genes. Hydroxymethylation levels were significantly more elevated in the neoplastic samples, particularly within satellites and complex repeats, likely related to structural functions. Additionally, our analysis also revealed distinct methylation and activity patterns in retrotransposons, providing additional insights into bivalve neoplastic processes. Altogether, these findings contribute to understanding the epigenetic dynamics of bivalve neoplasias and shed light on the roles of DNA methylation and hydroxymethylation in tumorigenesis. Understanding these epigenetic alterations holds promise for advancing our broader understanding of cancer epigenetics.

**Supplementary Information:**

The online version contains supplementary material available at 10.1007/s00438-024-02148-z.

## Introduction

Transmissible cancers defy all conventional thinking, as they involve neoplastic cells spreading between individuals beyond their original host (Murchison 2008) either through direct contact, as in the canine venereal tumour or the Tasmanian devil facial tumour (Murgia et al. 2006; Murchison et al. 2012) or by the absorption of cells freed to the media, as in hemic neoplasia in bivalves (Metzger et al. 2015, 2016; Bruzos et al. 2023; Hart et al. 2023). Nowadays, this phenomenon extends to, at least, six bivalve species (Metzger et al. 2015; Hart et al. 2023), among which, the common cockle, *Cerastoderma edule*, exhibits two transmissible cancer lineages, differentiated by cytological and genomic features (Bruzos et al. 2023). These neoplasias are evolutionary relics, accumulating mutations over time (Bruzos et al. 2023; Hart et al. 2023) and thus constitute unique models of tumour dynamics, metastasis and evolution (Garcia-Souto et al. 2022). Beyond mere scientific interest these neoplasias also have ecological and economic impacts, provoking mass mortalities in bivalve beds, especially amid climate change phenomena (Bramwell et al. 2021).

Our understanding of the epigenetic mechanisms underlying the evolution of bivalve transmissible neoplasia (BTN) remains unexplored (Bruzos et al. 2023; Hart et al. 2023). In vertebrates, DNA methylation in cancer cells undergoes notable alterations, often resulting in widespread hypomethylated genomes (Feinberg et al. 2006), although certain promoters experience abnormal hypermethylation, leading to inappropriate gene silencing (Herman et al. 1998; Feinberg et al. 2006; Dumitrescu and Verma 2012). This drives a transcriptome reprogramming wherein some genes are activated, typically proto-oncogenes, while others are silenced, usually tumour suppressor genes (Feinberg et al. 2006; Baylin and Jones 2011). Altered epigenetic profiles may also lead to Single Nucleotide Variation (SNV), as methylated cytosines are prone to transitioning to thymine, and larger structural mutations, given that, reduced methylation in centromeric heterochromatin is linked to non-reciprocal translocations (Dumitrescu and Verma 2012).

Levels of genomic methylation of adult bivalve tissues, around 20%, are much lower than the approximately 50% found in mammals (Sun et al. 2014; Gavery and Roberts 2017). Although promoter methylation seems to be inconsequential in bivalves, intragenic methylation—within exons and introns—in bivalves enhances gene transcription, particularly during larval development (Riviere et al. 2013; Lim et al. 2021). Intriguingly, reductions in methylation do not always coincide with a significant decrease in transcription, adding complexity to our understanding of these regulatory processes (Dixon and Matz 2022).

Intermediate products in the 5-methylcytosine (5mC) demethylation, among which 5-hydroxymethylcytosine (5hmC) is the most prevalent (Rasmussen and Helin 2016), may also hold further functional regulatory functions (Plongthongkum et al. 2014; Wu and Zhang 2015). 5hmC accumulates in the vertebrate central nervous system due to the oxidation of 5mC catalysed by ten-eleven translocation dioxygenases (Kinde et al. 2015) at ranges of *circa* 13% (Wen et al. 2014). Impairment of 5hmC levels is a recurrent hallmark of cancer in hematopoietic malignancies (Jin et al. 2011; Pronier et al. 2011; Rasmussen and Helin 2016). This adds an additional layer of complexity to the interplay between epigenetic signatures in the dynamic regulation of gene expression under normal and altered cell states.

Here, we conduct a comprehensive study of the epigenomic profiles of healthy and BTN lineages in *C. edule* by means of DNA methylation and hydroxymethylation analyses, shedding light on the intricate interplay between these epigenetic modifications and the transcriptome.

## Results

### BTN in cockles concurs with genomic hypomethylation

**Fig. 1:**
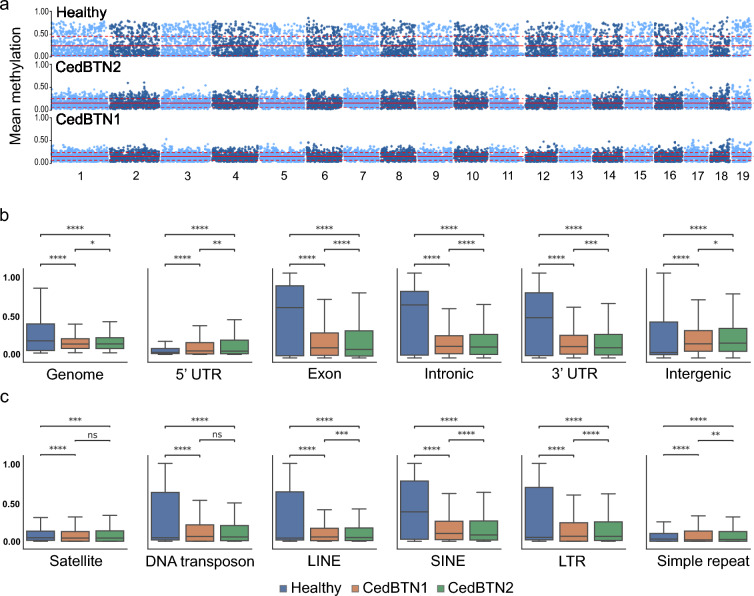
Whole genome methylation analysis in cockles. **a** Distribution of CpG methylation on 100 kb windows across the chromosomes of *C. edule* for a healthy specimen (ENCE17_3572) and two pure neoplastic haemolymphs of CedBTN1 (PACE20_537) and CedBTN2 (ENCE21_1202). Genomic methylation means are marked by red lines; red dashed lines indicate ± standard deviations. Boxplots displaying methylation levels of **b** gene-related features and **c** main repeat categories in the same specimens. *p* value annotation legend for a two-sided Mann–Whitney–Wilcoxon test: ns: not significant; * 1.00e−02 < *p* ≤ 5.00e−02; ** 1.00e−03 < *p* ≤ 1.00e−02; *** 1.00e−04 < *p* ≤ 1.00e−03; **** *p* ≤ 1.00e−04

In an initial analysis we compared whole genome methylation landscapes between a healthy (ENCE17_3572) and two neoplastic *C. edule* specimens (PACE20_537 and ENCE21_1202). Overall, CpG methylation was low for all the specimens. Most notably, the healthy specimen exhibited a higher genomic methylation (mean = 24.10%, SD = 21.37%, depth = 14.72) than the neoplastic haemolymph (PACE20_537: mean = 14.61%, SD = 8.43%, depth = 7.54, ENCE21_1202: mean = 15.55%, SD = 9.67%, depth = 13.02) (Fig. 1a; Supplementary Table 1). This hypomethylation trend, while occurring within the context of the inherently low CpG methylation levels characteristic of bivalves, is a hallmark shared by various cancer types in vertebrates, which alludes to the pivotal role that DNA methylation may play in the process of neoplastic transformation of cockles. Focusing on functional features (Fig. 1b) most gene parts (exons, introns and 3′-UTR) displayed higher methylation levels than 5′ UTR and intergenic regions of healthy specimens (Fig. 1b). We also observed higher methylation values for some repeats within the healthy specimen genome (Fig. 1c). In contrast, in the neoplastic samples most features of the genome were hypomethylated, being only the intergenic CpG more methylated than those of the healthy specimens (Fig. 1b, c). The healthy cockle showed an asymmetric CpG methylation and broader distributions for some of these features (Fig. 1b, c), while in the neoplastic samples a more homogeneous distribution across all genome features was detected.

In an effort to create a minimal predictor model for CpG methylation distribution, we conducted stepwise Akaike’s information criterion (AIC) regressions. For the healthy specimen (ENCE17_3572), we found positive correlations with gene content, CpG islands, and 5hmC levels. Conversely, indeterminations (that is, blocks of 500 consecutive “N”s introduced between contigs during the scaffolding of this reference genome), CG content, and tandem repeats negatively correlated with CpG methylation, with CG content being the strongest predictor. In the neoplastic samples (ENCE21_1202 and PACE20_537), the gene content and CpG islands were the main positive influencers on CpG methylation, while CG content had a negative impact.

Immunostaining against 5mC on mitotic metaphases of healthy and neoplastic (Supplementary Fig. 1) specimens revealed low and homogeneously distributed 5mC signals along most regions of the chromosomes, with no traces of over-methylated regions. However, in neoplastic interphase nuclei, a significant reduction in signal accumulation was evident compared to the healthy counterparts, aligning with the patterns observed in the entire genome methylome data.

### Impaired DNA methylation contributes to the neoplastic process

**Fig. 2:**
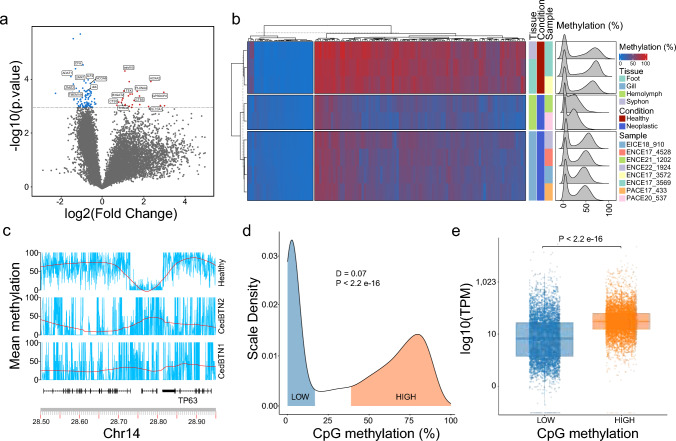
Intragenic methylation in cockles. **a** Volcano plot illustrating differences in intragenic methylation between healthy and neoplastic CedBTN1 tissues. The *X*-axis represents the log2(fold change) between tumour and non-tumour states, while the *Y*-axis displays the *t* test *p* values for the comparison. The horizontal bar indicates a *p* value threshold of 0.05. Over-methylated genes are highlighted in red, and under-methylated genes are shown in blue. **b** A dual hierarchical clustered heatmap presenting intragenic methylation across the cohort’s 114 differentially methylated genes (columns) in the analysed samples (rows). Smoothed kernel density charts comparing all genes methylation are on the side. **c** DNA methylation profiles across CpGs on a section of Chr14 encompassing three genes for the designated samples, namely ENCE17_3572 (healthy), PACE20_537 (CedBTN1) and ENCE21_1202 (CedBTN2). Interpolation curves are depicted in red, with 99% confidence intervals shaded. **d** Methylation level density plots across genes in the healthy ENCE17_3572 specimen. **e** Boxplots of gene expression levels of low and highly methylated genes in the genome of the healthy sample ENCE17_3572

To investigate the potential impact of DNA methylation on gene expression within the neoplastic process, we calculated fold changes and performed *t* tests to identify differentially methylated genes in healthy and neoplastic samples, accounting for all healthy (*n* = 3) and CedBTN1 neoplastic (*n* = 5) sequenced tissues. This analysis revealed hypomethylation in numerous genes in the neoplastic specimens, with some limited genes displaying increased methylation (Fig. 2a; Supplementary Table 2). A hierarchical clustering analysis of gene-wise methylation patterns across all samples unveiled three distinct groups: non-tumoral tissues, gills affected by CedBTN1 and pure neoplastic haemolymphs (Fig. 2b). Most notably, among the neoplastic-specific up-methylated genes were *NOTCH4* (Chr2:30616699–30617612) and *HOXA2* (Chr6:22902205–22919504), both consistently up-methylated in the neoplastic genomes. Our analysis also revealed that methylation can be gene-specific rather than affecting broader genomic regions. For instance, on Chromosome 14, three genes lie within a short region (Fig. 2c). Among them, *TP63*, known for encoding a p53 transcription factor with a tumour suppressor role, showed reduced methylation in the tumoral lineages. Interestingly, an adjacent unannotated gene exhibited the opposite trend, being up-methylated in tumoral samples while down-methylated in healthy specimens.

We then aimed to explore the impact of CpG methylation on the transcriptome of both healthy and neoplastic genomes, with a focus on the samples ENCE17_5274 (healthy), PACE20_537 (CedBTN1) and ENCE21_1202 (CedBTN2, Supplementary Table 3). Gene methylation profiles in healthy specimens showed a distinct bimodal distribution, as confirmed by a Hartigan’s dip test (Fig. 2d; *D* = 0.07; *p* value <0.05), comprising a group of genes with low methylation levels (2.52%; SD = 0.80%) and the other displaying much higher methylation values (61.66%; SD = 22.80%). An examination of the available RNAseq data for this sample revealed that the hypomethylated genes had reduced expression levels compared with those exhibiting hypermethylation (Fig. 2e). Furthermore, a Pearson correlation highlighted a moderate and positive connection between the proportion of intragenic methylated CpGs and their corresponding transcription levels (*p* = 0.41; *p* value <0.05, Supplementary Fig. 2). Accordingly, and albeit their global tendency towards genomic hypomethylation, the two neoplastic samples displayed the same patterns of differentially methylated genes (Fig. 2b). This observation was coupled with equally significant direct correlations between intragenic CpG methylation and their transcription levels, of *p* = 0.42 (*p* value < 0.05, Supplementary Fig. 2) for CedBTN2 and *p* = 0.34 (*p* value <0.05, Supplementary Fig. 2) for CedBTN1. Thus, our findings corroborate the role of intragenic CpG methylation in modulating gene expression in both tumoral and healthy genomes, suggesting its potential involvement in the neoplastic process.

### CpG hydroxymethylation displays a non-random distribution

**Fig. 3:**
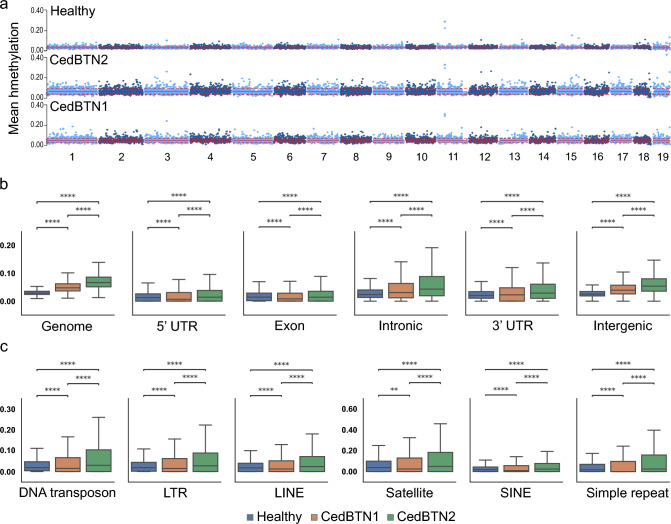
Whole genome hydroxymethylation analysis. **a** Distribution of CpG hydroxymethylation on 100 kb windows across the chromosomes of *C. edule* for three samples, a healthy specimen (ENCE17_3572) and two pure neoplastic haemolymphs of CedBTN2 (ENCE21_1202) and CedBTN1 (PACE20_537). The mean level of genomic hydroxymethylation is marked by red lines; red dashed lines indicate ± standard deviation. Boxplots displaying hydroxymethylation levels of **b** gene-related features and **c** the main repeat categories in the same specimens. *p* value annotation legend for a two-sided Mann–Whitney–Wilcoxon test: ns: not significant; * 1.00e−02 < *p* ≤ 5.00e−02; ** 1.00e−03 < *p* ≤ 1.00e−02; *** 1.00e−04 < *p* ≤ 1.00e−03; **** *p* ≤ 1.00e−04. Of note, the *Y*-axis scales have been adjusted to align features with comparable hydroxymethylation levels

In the cockle healthy genome (ENCE17_3572) the 5hmC levels, only 2.83%, were significantly lower on average than those of 5mC. In contrast, in tumoral genomes 5hmC levels reached 4.60% for CedBTN1 (PACE20_537) and 6.29% for CedBTN2 (ENCE21_1202), values significantly higher than those observed in healthy samples (Fig. 3a; Supplementary Table 4). 5hmC remained stable across various functional regions, although consistently higher in both neoplastic lineages (Fig. 3b, c). Linear regression models revealed consistent trends across all samples and conditions: hydroxymethylation increased with tandem repeats and CpG islands but decreased with GC content and genes.

Most notably, 5hmC was concentrated on satellite DNAs in both healthy and neoplastic samples, including those in intragenic repeats. An example of this is CLPP (Chr2:32138969–32158982), featuring an intronic CpG-island with 26 bp tandem repeats showing notably higher hydroxymethylation levels compared to its genomic context (Supplementary Table 4). While not conclusively demonstrated, we speculate that this increased 5hmC density at these tandems may be linked to distinct chromatin condensation or secondary DNA structures related to chromatin accessibility. However, a remarkable exception was found in CedS4, the major satellite DNA occupying the heterochromatic regions in the healthy lineage (Bruzos et al. 2023), which was found largely depleted of CpG modifications (Supplementary Table 5), as also observed in the methylation and hydroxymethylation desert at the end of Chromosome 11, primarily composed of this satellite (Figs. 1a, 3a). Contrary to 5mC, we did not find a bimodal distribution of 5hmC across the gene features, nor did we observe a relevant impact on the gene expression of their individual genes (Supplementary Table 6).

### Epigenetic signatures of retrotransposons

**Fig. 4:**
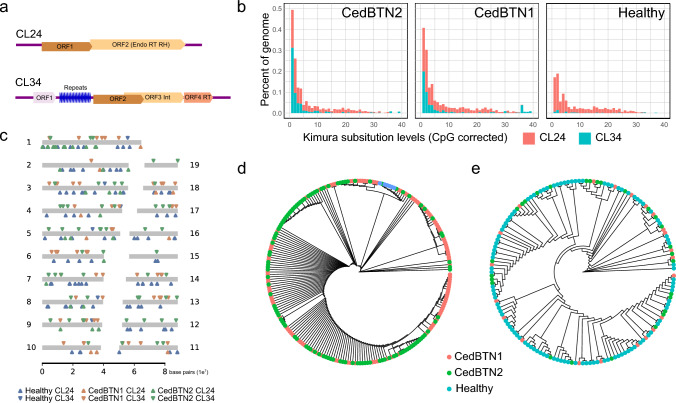
Genomic features of selected retrotransposon in healthy and neoplastic cockles. **a** CedCL34 is a ~7.5 kb TE with four Open reading Frames (ORFs), including integrase (Int) and reverse-transcriptase (RT), separated by internal tandem repeats, while CedCL24, spanning 6.3 kb, harbours a multifunctional Endonuclease (Endo), RT and Ribonuclease H (RH) single ORF. **b** Divergence plots of CedCL34 and CedCL24 across the genomes of the aforementioned samples, including a healthy specimen (ENCE17_3572) and two neoplastic CedBTN1 (PACE20_537) and CedBTN2 (ENCE21_1202) haemolymphs, revealed an expansion of CedCL24 in the three genomic lineages, while CedCL34 was found abundant solely in the neoplastic genomes. **c** Ideogram depicting the chromosomal insertion points of CedCL34 and CedCL24 in the *C. edule* reference healthy (ENCE17_3572), CedBTN1 (PACE20_537) and CedBTN2 (ENCE21_1202) genomes. **d** Neighbour joining phylogeny based on 228 full length CedCL34 copies isolated from the genomes of the healthy (ENCE17_3572, 6 copies), CedBTN1 (PACE20_537, 113 copies) and CedBTN2 (ENCE21_1202, 109 copies) genomes. **e** NJ tree of 154 full length CedCL24 elements recovered from the healthy (ENCE17_3572, 104 copies), CedBTN1 (PACE20_537, 22 copies) and CedBTN2 (ENCE21_1202, 28 copies) genome reconstructions

Upon revisiting the RepeatExplorer2 output in Bruzos et al. (2023), we identified two potential retrotransposable elements, CedCL34 (≈7.5 kb) and CedCL24 (6.2 kb), present in both healthy and neoplastic *C. edule* specimens. CedCL34, having internal 61 bp direct repeats splitting them into two segments, contained four putative Open Reading Frames (ORFs, Fig. 4a). CedCL34_ORF3 showed similarity to bivalve integrase/recombinase xerD enzymes. Meanwhile, CedCL34_ORF4 harboured a reverse transcriptase domain linked to DIRS1 family retroelements (Poulter and Goodwin 2005). CedCL24, on the other hand, contained two putative ORFs, with CedCL24_ORF2 associated with endonuclease, reverse transcriptase, and ribonuclease H functions and classified as a LINE1 retrotransposon (Fig. 4a). CedCL34 was found selectively expanded in the neoplastic lineages, displaying low sequence variability among copies and accounting for 0.93% and 0.65% in, respectively, CedBTN1 and CedBTN2, while less abundant in the healthy genome, reaching a coverage of merely 0.01% (Fig. 4b) and only six complete copies detected (Fig. 4c). Leveraging long-read sequencing, we characterized the mobilization events of CedCL34 across both BTN genomes, detecting 54 and 60 insertions in, respectively, CedBTN2 (ENCE21_1202) and CedBTN1 (PACE20_537) genomes (Fig. 4c). These BTN insertions exhibited a distinct pattern, stacking multiple tandem repeats of the transposon, some of which were rearranged, in a satellite-like configuration, a distribution absent in the healthy sample analysed in this study (Supplementary Figs. 3, 4). Importantly, these insertions revealed no common events, aligning with expectations from lineages of distinct genetic origins.

Similarly, CedCL24 also exhibited limited sequence diversity among copies, yet unlike CedCL34, it was conspicuously abundant in the healthy genome (Fig. 4b). It displayed an average normalized coverage of 0.30%, 0.33%, and 0.38% for the healthy, CedBTN1 and CedBTN2 genomes, respectively. Remarkably, searches within the healthy genome unveiled only six full-length copies of CedCL34, signifying its scarcity, while CedCL24 was more widely distributed across their chromosomes (Fig. 4c). A phylogenetic analysis of the retrieved CedCL34 sequences revealed a distinct cluster encompassing all healthy copies, whereas CedBTN1 and CedBTN2 copies intermingled throughout the phylogenetic tree (Fig. 4d). This suggests a divergent evolutionary path for CedCL34 between healthy and tumoral genomes, presumably stemming from post-lineage radiation. Conversely, a phylogenetic examination of 154 full-length copies of CedCL24 did not reveal any exclusive clustering of variants from different lineages (Fig. 4e). Utilizing long-read sequences, we detected 31 and 13 insertion points for CedCL24 in, respectively, CedBTN2 (ENCE21_1202) and CedBTN1 (PACE20_537) samples, with no shared events between them (Fig. 4c). Noteworthy was the prevalence of head-to-tail dimeric repeats of the full CedCL24 length element found in both BTN samples, a configuration conspicuously absent in the healthy specimen ENCE17_3572 genome, for which no additional insertions beyond those present in the reference genome could be detected (Supplementary Figs. 5, 6). These findings imply an early expansion of CedCL24 during *C. edule*’s evolutionary history, likely preceding the divergence of the first tumoral lineage.

**Fig. 5:**
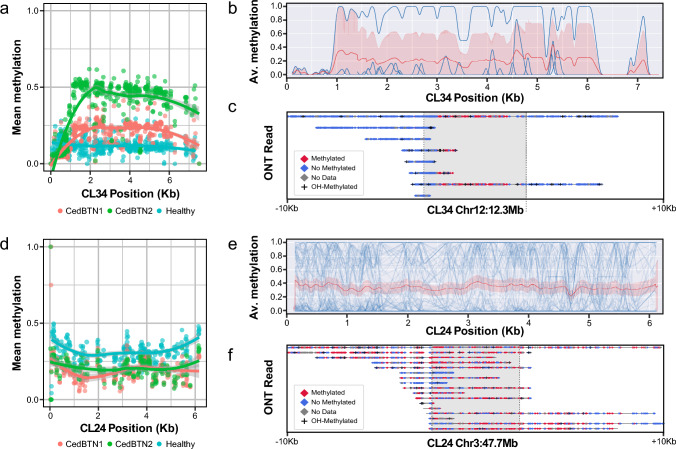
CpG methylation profiles of CedCL34 and CedCL24 retrotransposons on the genomes of healthy and neoplastic *C. edule* specimens. **a** CpG methylation levels across the consensus sequence of CedCL34 on the CedBTN1 (PACE20_537) and CedBTN2 (ENCE21_1202) samples than in the healthy specimen ENCE17_3572. **b** CpG methylation content in the six complete CedCL34 copies from ENCE17_3572, showing predominantly down-methylation (5/6). Data was smoothed using 20 bp rolling average windows. The mean and 95% confidence intervals are highlighted in red. **c** Epigenetic profiling of CpG sites per nanopore read mapped on CedCL34 loci at Chr16:9885244–9892715 in the healthy sample ENCE17_3572, revealing pronounced over-methylation compared to its genomic neighbourhood. **d** CedCL24 mean methylation levels were slightly higher than the average of the three genomes tested. **e** CpG methylation profiling for each full-length CedCL24 element from healthy specimen ENCE17_3572. Mean and 95% confidence intervals highlighted as before. **f** By-read methylation patterns example on the full-length CedCL24 element at Chr3:47751866–47757975 in the genome of healthy specimen ENCE17_3572. ONT = Oxford Nanopore Technologies

Our analysis revealed that the level of CpG methylation on CedCL34 was relatively low in the healthy sample (ENCE17_3572, mean = 11.75%, SD = 4.47), moderately higher in CedBTN2 (ENCE21_1202, mean = 18.88%, SD = 8.58%) and significantly increased in CedBTN1 (PACE20_537, mean = 37.44%, SD = 16.80%) (Fig. 5a). The availability of a chromosome level assembly for *C. edule* allowed us to conduct element-wise specific methylation analysis for the healthy sample. There, most of the complete CedCL34 copies (5/6) presented low CpG methylation levels (Fig. 5b), being the solely exception the element at Chr16:9885244–9892715, whose methylation levels were actually much higher when compared to neighbouring regions (Fig. 5b, c). Examining the available RNAseq data from the neoplastic specimens PACE20_537 and ENCE21_1202, it was evident that both CedCL34_ORF3 and CedCL34_ORF4 showed higher transcripts per million (TPM) values in the neoplastic samples (CedBTN1: CedCL34_ORF3 = 2130.50 TPM, CedCL34_ORF4 = 6237.98 TPM; CedBTN2: CedCL34_ORF3 = 1801.91 TPM, CedCL34_ORF4 = 2838.41 TPM) than in the healthy specimen (ENCE17_3572, CedCL34_ORF3 = 103.06 TPM; CedCL34_ORF4 = 5.23 TPM). This elevated expression in neoplasias positively correlated with higher methylation levels compared to the healthy specimen, likely also influenced by the increased number of transposon copies in tumours, either through basal expression or specific loci hyperactivity.

CedCL24, on the other hand, showed CpG methylation values slightly higher than the genome averages on both healthy (mean = 32.14%. SD = 6.78%, depth = 4439), CedBTN1 (mean = 19.58%, SD = 9.59%, depth = 2824) and CedBTN2 (mean = 20.96%, SD = 8.29%, depth = 5561) genomes (Fig. 5d). This was coupled to high expression values of *CedCL24_ORF2* in the three lineages (CedBTN1 = 2268.52 TPM, CedBTN2 = 2465.91 TPM, healthy = 840.02 TPM), which also supports the idea of DNA methylation mediating in the activity of these transposable elements as an activation epigenetic landmark. The availability of long read sequences allowed us to characterize the methylation status of all full-length loci in the healthy specimen, which revealed a variety of seemingly stochastic methylation patterns (Fig. 5e), from mostly non-methylated to moderately methylated, as it is the case for the locus at Chr12:12355140 (Fig. 5f), and highly methylated, with consistent patterns between reads mapping on the same locus.

## Discussion

Despite the constraints on sample size in this study, primarily attributed to difficulties associated with obtaining CedBTN samples of sufficient purity and quantity, our results provide significant insights into the epigenetic landscape governing the transmissible neoplasias of *C. edule*, thereby elucidating the intricate dynamics of DNA methylation in these bivalves. Despite their evolutionary divergence from vertebrates, our findings unveil compelling parallels in the realm of DNA methylation and its influence on gene expression within both neoplastic and healthy specimens.

While occurring within the context of the inherently low CpG methylation levels characteristic of bivalves (Sun et al. 2014; Gavery and Roberts 2017; Garcia-Souto et al. 2017), the pervasive genomic hypomethylation observed in the neoplastic haemolymphs is a hallmark shared by various cancer types (Ehrlich 2009; Hanahan and Weinberg 2011). This epigenetic reprogramming may allude to the pivotal role that DNA methylation may play in the process of neoplastic transformation of cockles, by disrupting the homeostatic balance of chromatin, causing it to become either aberrantly restrictive or permissive (Flavahan et al. 2017). Particularly compelling was the connection between intragenic methylation and increased gene expression, in concordance with previous findings in molluscs (Gavery and Roberts 2014; Wang et al. 2014). Of particular significance is the observation that, although the impact of this epigenetic mark contrasts fundamentally with its repressive function in vertebrates, its role in tumorigenesis appears consistent by the facilitation of the selective activation of potential oncogenes, thereby substantiating its involvement in the neoplastic process as a positive regulator of gene expression. Nevertheless, it is also important to note that the regulatory function of DNA methylation in invertebrates is not yet fully understood. While intragenic methylation has been found to positively correlate with gene expression in both invertebrates and plants (Gavery and Roberts 2010, 2013, 2014; Wang et al. 2014), capacitating the activation of housekeeping genes (Gavery and Roberts 2014), the extent of its involvement in modulating gene expression is not entirely clear (Feng et al 2010). Recent findings suggest that the relationship between methylation and transcription is not strictly linear, since deep differences in transcriptomes have been observed in various cell types, despite posing similar overall methylation profiles (Gatzmann et al. 2018; Harris et al. 2019; de Mendoza et al. 2020). Furthermore, removal of methylation through the targeted inhibition of *DNMT1* does not significantly alter gene expression (Bewick et al. 2017). These studies support the idea that changes in intragenic DNA methylation of invertebrates are neither necessary nor sufficient to induce transcriptional changes, suggesting the interplay of DNA methylation with other epigenetic mechanisms in regulating their gene expression as a capacitor (Gavery and Roberts 2017).

Despite this prevailing trend towards genomic hypomethylation, our analysis also revealed genes exhibiting up-methylation within the neoplastic genomes, yet another common characteristic to vertebrate cancer, in which some genes evade the general trend (Hanahan and Weinberg 2011). This includes some genes with known oncogenic functions in vertebrates, such as *NOTCH4* or *HOXA2* (Ohnemus et al. 2001; Shimizu et al. 2002), which consistently exhibited heightened methylation in the neoplastic CedBTN genomes. *NOTCH4* functions as a membrane receptor in regulating cell-fate determination, including cell differentiation, proliferation, and apoptosis in vertebrates (Shimizu et al. 2002) and it is overexpressed in various types of cancer, including breast and colorectal cancer (Nagamatsu et al. 2014; Wu et al. 2018). Similarly, *HOXA2* is part of the homeobox transcription factor family involved in cell differentiation during embryo development (Ohnemus et al. 2001), acting as an oncogene in a variety of tumours (Simeone et al. 1990). These findings suggest that DNA methylation might play a role in the neoplastic process of *C. edule*, and that, despite the marked different regulatory functions of CpG methylation, the epigenetic signatures on CedBTN mirror the patterns observed in vertebrate cancers, by capacitating the transcription of oncogenes. Moreover, our analysis emphasizes a precise, gene-specific targeted DNA methylation rather than sporadic epigenetic alterations operating in broader genomic regions, as exemplified by the distinct 5mC profiles of contiguous genes.

Our results underscore a non-random distribution of hydroxymethylation throughout the analysed genomes, with neoplastic specimens displaying slightly higher levels. In this regard, a particularly intriguing finding is the pronounced concentration of 5hmC within satellite DNAs, especially for those found within intragenic repeats. This suggests a potential link of 5hmC in maintaining chromatin condensation or secondary DNA structures, likely related to regulatory functions to some extent, despite the evident lack of correlation with gene expression. 5hmC is recognized as a stable and relatively abundant epigenetic modification within the vertebrate genome (Rasmussen and Helin 2016) and plays a significant role in the repression of genes involved in embryonic development, cell fate determination and cellular differentiation (Plongthongkum et al. 2014; Wu and Zhang 2015). Additionally, 5hmC is actively involved in the regulation of neuronal development and synaptic plasticity within the neural system (Wen et al. 2014). While data in non-vertebrates is more limited, recent studies highlight the concentration of 5hmC in regulatory regions appears consistent with its function in developmental processes (Skvortsova et al. 2022). In altered or pathological states, changes in CpG hydroxymethylation have been associated with various diseases. For instance, global loss of hydroxymethylation in CpGs is often observed in hematopoietic cancer cells, and this loss is linked to abnormal DNA methylation patterns and gene expression changes (Jin et al. 2011; Pronier et al. 2011; Rasmussen and Helin 2016). Thus, our findings hint at a potential structural role for hydroxymethylation in the tumours of *C. edule* and open new research avenues.

The notable absence of epigenetic modifications, either 5mC or 5hmC, within CedS4, the major satellite DNA composing the heterochromatic regions of the healthy lineage, hints at a distinct regulatory mechanism or role for CedS4 in upholding its functions. As the primary constituent of healthy cockle heterochromatin, CedS4 likely assumes a function in maintaining the structural integrity and packaging of this region, paralleling certain satellite DNAs in humans that participate in repressing transposons and ensuring genome stability (Gent and Dawe 2012; Hall et al. 2012). Thus, CedS4 might be subject to a different or specialized epigenetic regulation (i.e. histone variants), if any, rendering it refractory to CpG modifications. Previous studies in the major satellite DNA composing the heterochromatin of another bivalve, *Spisula subtruncata*, revealed similarly low methylation levels (García-Souto et al. 2017), implying that these satellite DNAs may exhibit conserved epigenetic patterns across bivalves.

The distinct behaviours of CedCL34 and CedCL24 in the analysed samples suggest different evolutionary histories. CedCL34 appears to be active at present in both CedBTN lineages, as evidenced by its selective expansion and low sequence variability among copies. This could indicate a recent burst of retrotransposon activity specifically in the tumoral lineages with no chance of accumulating mutations since. In contrast, CedCL24 displays limited sequence diversity among copies and abundance in both healthy and neoplastic genomes, supporting an early expansion during the evolution of *C. edule*, possibly prior to the divergence of the first tumoral lineage. The inference of these scenarios is based on the preservation of sequence integrity within the elements, as observed in previous studies (Plohl et al. 2010; Thakur et al. 2021). Of note, our long-read analysis unveiled a predominant occurrence of aberrant integrations of both transposable elements within the two CedBTN lineages as head-to-tail tandem repeats. These insertion loci bear similarity to those found in other bivalves such as the retrotransposon family biv-TRIM in *Donax trunculus* (Šatović et al. 2019).

The differential expression of CedCL34 and CedCL24 in the studied lineages further supports their distinct activity profiles. CedCL34 is predominantly expressed in the tumoral lineages, with notably higher expression levels, while CedCL24 shows high expression levels in all lineages, albeit at a lower level in the healthy specimen than in both neoplastic samples. It is worth noting that the increased number of transposon copies along the tumoral genomes could potentially bias this analysis, as more copies may lead to a higher probability of transcriptional activity. These findings are in line with previous analyses in *Mya arenaria* BTN (MarBTN), which revealed similar results with the activation of a Steamer-like elements (Arriagada et al. 2014) and a larger number of copies in the neoplastic samples (Hart et al. 2023), although further analysis in other species revealed that the amplification of retrotransposons is not essential in the development of BTN (Metzger et al. 2016).

The differences in methylation levels among these transposons provide intriguing insights into the role of DNA methylation in the context of transposable elements (TE). In vertebrates, DNA methylation has long been recognized as a pivotal mechanism for suppressing TE activity, preventing their disruptive effects on gene expression and genomic integrity (Goodier 2016). This recognition forms the basis for the assumption that increased DNA methylation should concur with the inhibition of TEs, as otherwise proposed for bivalves (Gavery and Roberts 2014). However, our findings challenge this paradigm and suggest that the relationship between DNA methylation and TE mobilization is actually the opposite to that of vertebrates and, as intragenic CpG methylation, promotes TE activity. Indeed, our results revealed higher DNA methylation within the CedCL34 elements in both neoplastic genomes, where these elements are significantly more expanded and active when compared to the healthy genome. The methylation profiles of CedCL24 also support this notion, as these exhibit high methylation levels, as well as high transcriptional activity across all three lineages. This leads us to contemplate a role for DNA methylation in regulating TEs in bivalves and their neoplasias, possibly as a capacitor of their mobilization, analogous to that for genes (Gavery and Roberts 2014).

An alternative explanation is that the higher DNA methylation in the neoplastic genomes does represent an epigenetic response to silence these TEs as a means to control their activity, and thus the observed higher expression would be attributed to a limited number of non-methylated and active source elements (Gavery and Roberts 2014). This response might be triggered by the need to maintain genome stability or to suppress excessive TE mobilization on the neoplastic genomes. In this scenario, DNA methylation could act as a dynamic switch, fine-tuning TE activity, which can also vary in different genomic contexts. Nevertheless, further refined analyses are warranted to confirm these observations and determine the precise relationship between methylation and the activity of these transposable elements.

Overall, our research underscores the distinctive nature of epigenetic regulation in *C. edule*, particularly in the context of its transmissible neoplasias. The observed global hypomethylation in neoplastic specimens, the intricate interplay between DNA methylation and gene expression and the differential methylation patterns exhibited by genes and retrotransposons collectively shed light on the neoplastic process in these bivalve species. While our study reveals intriguing similarities with vertebrate systems, it also underscores the unique epigenetic mechanisms operative in *C. edule*. This contribution enhances our comprehension of epigenetics in non-model organisms and its implications for cancer in marine life. Further epigenetic studies, including a larger dataset of healthy and neoplastic samples, are warranted to obtain a more robust insight into the complex interplay of epigenetic mechanisms in bivalves and their neoplasias.

## Methodology

### Sampling and diagnosis of disseminated neoplasia

Wildlife specimens were sampled from natural beds and housed at the University of Santiago de Compostela Aquarium facilities until further analysis. Diagnosis of disseminated neoplasia was carried out using standard cytological methods based on haematoxylin–eosin-stained haemolymph extensions, as described by Carballal et al. (2015). This approach enabled the characterization of disease severity, cytological type and stage for each animal. The intensity of neoplasia was quantified by counting tumoral and non-tumoral haemocytes classifying the affected specimens into four categories: N0 (no tumoral hemocytes), N1 (early affectation, 0–15%), N2 (medium affectation, 15–75%), and N3 (severe affectation, >75%).

DNA and RNA extractions from tissues were performed using the All Prep DNA/RNA Kit (QIAGEN), with some modifications. In brief, the proteinase digestion was supplemented with 10% β-mercaptoethanol to reduce nuclease activity. Following this, the digestion mixture was supplemented with 400 µL of 10% SDS, followed by gentle inversion and a 20 min incubation at 70 ºC. Subsequently, 500 µL of 5 M CH_3_COOH was introduced and after inverting the tubes, the samples were kept on ice for 20 min to allow precipitation of SDS and lipopolysaccharides. The supernatant was processed following the subsequent steps as in the manufacturer’s instructions. The extracted DNAs and RNAs were outsourced to Macrogen for Illumina paired-end DNA sequencing. DNA sequencing was conducted on an Illumina NovaSeq 6000 platform using DNA TruSeq libraries (Illumina), resulting in 150 bp paired reads of 350 bp inserts with a final yield of 32.59 Gb and 36.59 for, respectively, ENCE21_1202H and PACE20_537H. Similarly, RNA-seq was performed on cDNA libraries (RNA TruSeq, Illumina) after ribosomal RNA cleavage with Ribo-zero (Illumina) and sequenced on an Illumina NovaSeq 6000, obtaining 150 bp paired-end reads with 350 bp insert sizes to a final yield of 68.91 Gb, 16.02 Gb and 16.66 Gb for ENCE17_3572F, ENCE21_1202H and PACE20_537H_RNA, respectively. DNA sequencing was utilized to perform nuclear and mitochondrial single nucleotide variant analysis to classify the corresponding samples into their specific neoplastic clones, namely CedBTN1 (neoplasia A) and CedBTN2 (neoplasia B) according to the diagnostic variants outlined by Bruzos et al. (2023). In brief, DNAseq were aligned against the reference genome with BWA (v0.7.17, Li and Durbin 2017), followed by sorting using samtools (v1.14, Danecek et al. 2021) and removal of duplicate reads using biobambam (Tischler and Leonard 2015). Variant calling was then conducted with GATK mutect2 (v4.1.4.1, GATK Team 2023) in “tumour-only” mode with default parameters. A total of 8 individuals were selected, including tissues from specimens obtained in Bruzos et al. (2023) and as provided in Table 1.

**Table 1:** Summary of the studied specimens

Individual	Neoplasia	Genetic lineage	Intensity	Tissue	Sampling points	Reference
ENCE17_3572	No	NA	N0	Foot	Noia, Spain	Bruzos et al. (2023)
ENCE17_3569	No	NA	N0	Foot, Siphon	Noia, Spain	This work
EICE18_910	A	CedBTN1	N3	Gills	Camariñas, Spain	Bruzos et al. (2023)
PACE17_433	A	CedBTN1	N3	Gills	Algarve, Portugal	Bruzos et al. (2023)
ENCE17_4528	A	CedBTN1	N3	Gills	Noia, Spain	Bruzos et al. (2023)
ENCE22_1924	A	CedBTN1	N3	Gills	Noia, Spain	This work
PACE20_537	A	CedBTN1	N3	Haemolymph	Algarve, Portugal	This work
ENCE21_1202	B	CedBTN2	N3	Haemolymph	Noia, Spain	This work

### Genome information and feature tracks

The reference assembly for *C. edule* (NCBI GenBank: GCA_947846245.1, Bioproject: PRJEB58149) was employed for downstream analysis (Bruzos et al. 2023). Genome feature tracks, encompassing genes, exons, UTRs and repeats, were retrieved from the same source (Bruzos et al. 2023). Introns were deduced by determining the complement of the exon track, achieved through a combination of BEDtools (v.2.26.0, Quinlan and Hall 2016) complementBed and intersectBed. Similarly, intergenic regions were isolated by finding the complement of genes using complementBed. For the identification of GC islands, Gcluster (Li et al. 2020) was utilized, and the results were transformed into bed annotation files using custom scripts. Satellite tracks were obtained using Tandem Repeats Finder (v4.09.1, Benson et al. 2020) at default parameters.

### Genome assembly and TE characterization

We analysed Illumina PE data from both tumoral lineages to characterize repetitive DNAs with RepeatExplorer2 (Novák et al. 2013). Subsequently, we identified transposable elements in either healthy or neoplastic lineages, for which we conducted ORF predictions using ORFfinder (Rombel et al. 2002), translated these into proteins and conducted homology searches against NCBI and Interproscan databases to infer their putative functions. The selected elements were compiled into a transposable element (TE) library, which served as input for two RepeatMasker utilities (Smit et al. 2022), calcDivergenceFromAlign.pl and createRepeatLandscape.pl, to generate interspersed repeat landscapes visualizing the relative abundance and nucleotide divergence of these elements out of Illumina data from healthy, CedBTN1, and CedBTN2 samples. We applied blastn scans to the *C. edule* reference genome and genome drafts derived from each of the purest neoplastic samples, as reconstructed with Flye (v2.9, Kolmogorov et al. 2019) with default parameters to recover full-length copies of the selected retrotransposons. Subsequently, multiple sequence alignments were performed using MUSCLE (v.5.1.0, Edgar 2022), and molecular phylogenetic analyses were conducted using the Neighbor-Joining algorithm implemented in Geneious (v11.0.9+11, Kearse et al. 2020). To trace transposon insertions, Nanopore reads were aligned to the *C. edule* reference genome (NCBI GenBank: GCA_947846245.1, Bioproject: PRJEB58149) using minimap2 v2.24 (Li 2018) and structural variation calling was performed utilizing sniffles v2.2 (Sedlazeck et al. 2018). Subsequently, additional filters were applied to the identified insertions, including criteria such as Support > 3 reads, PASS and PRECISE. The sequences of these filtered insertions were then subjected to BLAST analysis against the CL24 and CL34 consensus elements to identify potential insertions associated with these elements. Insertions exceeding 5 kb underwent further validation using Geneious v11.0.9+11 (Kearse et al. 2020).

### Long-read sequencing and whole-genome epigenetics

Non-fragmented DNAs were subjected to size selection using a SRE XS buffer (Circulomics) prior to end-prep repair (NEBNext kit, New England Biolabs) and long-read whole-genome sequencing libraries were obtained with an Oxford Nanopore sequencing-by-ligation kit (SQK-LSK109). Sequencing was conducted into MinION R9.4 (FLO-MIN106) flowcells in MinION sequencers controlled by the MinKNOW software (v18.12.09). Basecalling was conducted using Guppy (v6.0.1, Oxford Nanopore 2022a) with the high accuracy model. The final yields were 33.37 Gb, 2.44 Gb, 0.96 Gb, 16.68 Gb, 7.95 Gb, 5.64 Gb, 20.26 Gb and 59.94 Gb for, respectively ENCE17_3572F, ENCE17_3569F, ENCE17_3569S, EICE18_910B, ENCE17_4528B, PACE17_433B, ENCE22_1924B and ENCE21_1202H. The estimation of methylation and hydroxymethylation across all CpGs in the reference genome was performed with Megalodon (v2.5.0, Oxford Nanopore 2022b) using the neuronal network model remora-modified-bases dna_r9.4.1_e8 hac 0.0.0 5hmc_5mc CG 0. Estimates of 5mC and 5hmC on the satellite elements were conducted separately, using the consensus sequences for these elements as references.

Global epigenetic patterns were characterized by averaging DNA methylation and hydroxymethylation levels on CpG dinucleotides within 100 kb non-overlapping genomic windows. The fraction of each window occupied by gene features (UTR, exons, and introns), transposable elements, and other complex repeats, as well as CpG islands and putative transposable elements, was determined using intersectBed from BEDtools (Quinlan and Hall 2016). Additionally, CG and indetermination content was assessed using custom Python scripts. To select an appropriate linear model that best described the distribution of CpG methylation and hydroxymethylation with the less possible explanatory predictive variables, a stepwise selection procedure based on Akaike’s information criterion (AIC) was conducted with the package MASS, as implemented in R (Ripley et al. 2023). Differences between the normal and tumoral samples expressed as fold changes between windows or genomic features (i.e. genes) were calculated from the methylation tables obtained via Megalodon with gtools (v3.9.4) (Bolker et al. 2022). To determine the statistical significance of these differences, two-sided Mann–Whitney–Wilcoxon tests were conducted in Python using the libraries pyjanitor (v.0.24.0, Ma et al. 2018), scikit-learn (v.1.0.2, du Boisberranger et al. 2021), scipy (v.1.9.3, Virtanen et al. 2022), seaborn (v.0.11.2, Waskom 2021) and statannotations (v.0.5.0, Charlier et al. 2022). Furthermore, a principal component analysis was carried out on the samples using *prcomp* function from the stats package (v.3.6.2, Bolar 2022).

### Comparative transcriptomics

Gene expression levels based on the reference transcriptome for *C. edule* were assessed with SALMON (v1.10.1) (Patro et al. 2017) in R on the non-tumoral (ENCE17_3572) and two neoplastic samples (ENCE21_1202H, BTN1 and PACE20_537H, BTN2). Raw counts per gene were normalized to transcripts per million (TPM) to enable meaningful comparisons across samples. Pearson correlation tests (*α* = 0.05) were conducted to evaluate the relationship between methylation and transcription for each gene in each specimen.

### Cytogenetics and immunodetection

Mitotic chromosomes were obtained according to García-Souto et al. (2017). Healthy and neoplastic specimens were treated with colchicine (0.005%, 8 h) before dissection. Gills underwent hypotonic treatment in diluted seawater, followed by fixation in ethanol-acetic acid (3:1). Gill fragments were then disaggregated in acetic acid (60%) and placed on glass slides. Immunodetection of 5mC utilized mouse anti-5mC antibodies (Eurogentec) and FITC-conjugated goat anti-mouse antibodies (Sigma) as indicated by Covelo-Soto et al. (2014). Chromosomes were counterstained with DAPI (0.14 μg/mL in 2× SSC) and propidium iodide (PI: 0.07 μg/mL in 2× SSC), mounted with Vectashield antifade medium, and imaged using a Nikon Eclipse E800 microscope equipped with an epifluorescence system and a DS-Qi1Mc CCD camera controlled by NIS-Elements. Image processing was conducted using Adobe Photoshop.

### Supplementary Information

Below is the link to the electronic supplementary material.

Supplementary file1 (XLSX 5135 KB)

Supplementary file2 (FASTA 1119 KB)

Supplementary file3 (FASTA 1787 KB)

Supplementary file4 (PDF 13642 KB)

## Data Availability

All methylation and hydroxymethylation calling and RNAseq derived analysis were attached as supplementary data. Sequences and alignments for the transposable elements detected herein were also included as supplementary material. All short and long-read DNAseq and RNAseq fastq files derived from this project were uploaded to NCBI under the bioproject PRJNA1088420.
