# Supplementary material for: Unravelling epigenetic mechanisms in Cerastoderma edule genome: a comparison of healthy and neoplastic cockles
Source: Mol Genet Genomics. 2024 May 25;299(1):58. doi: 10.1007/s00438-024-02148-z (PMC11126487; doi:10.1007/s00438-024-02148-z)
Supplement: Supplementary file 4 — Supplementary file4 (PDF 13642 KB) [file 438_2024_2148_MOESM4_ESM.pdf]

Supplementary Figures

Unraveling epigenetic mechanisms in *Cerastoderma edule* genome: A comparison of healthy and neoplastic cockles. **Molecular Genetics and Genomics**

Alejandro Viñas-Feás[1], Javier Temes-Rodríguez[1], André Vidal-Capón[2], Samuel Novas[2], Jorge Rodríguez-Castro[1,3], Ana Pequeño-Valtierra[1,3], Juan José Pasantes[2], Jose MC Tubío[1,3,4,#], Daniel García-Souto[1,3,4,5,#,*]

[1]Genomes and Disease, Universidade de Santiago de Compostela. Centre for Research in Molecular Medicine and Chronic Diseases (CiMUS), Universidade de Santiago de Compostela, Santiago de Compostela, Spain.

[2]Centro de Investigación Mariña, Universidade de Vigo, Vigo, Spain.

[3]Instituto de Investigaciones Sanitarias de Santiago de Compostela (IDIS), Santiago de Compostela, Spain.

[4]Department of Zoology, Genetics and Physical Anthropology, Universidade de Santiago de Compostela, Santiago de Compostela, Spain.

[5]Department of Biological Sciences, School of Environment, Arts and Society, College of Arts, Sciences & Education (CASE), Florida International University, Miami, USA.

#These authors contributed equally

*Corresponding authorship

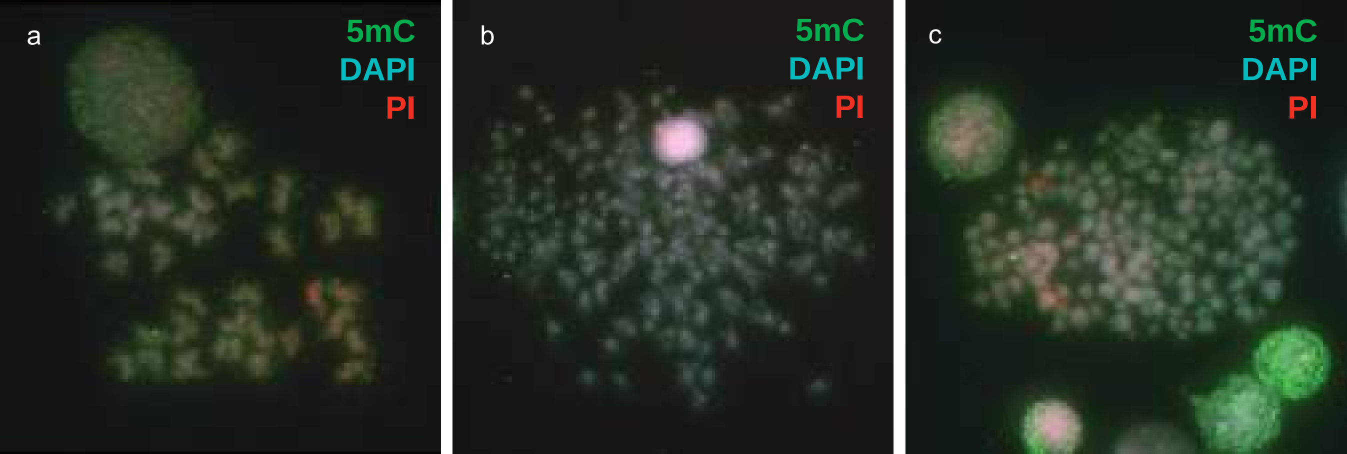
a
5mC
DAPI
PI
b
5mC
DAPI
PI
c
5mC
DAPI
PI

Supplementary Figure 1: Immunolocalization of 5-methylcytosine (5mC) in healthy (a) and neoplastic CedBTN1 (b) and CedBTN2 (c) metaphases of *C. edule*.

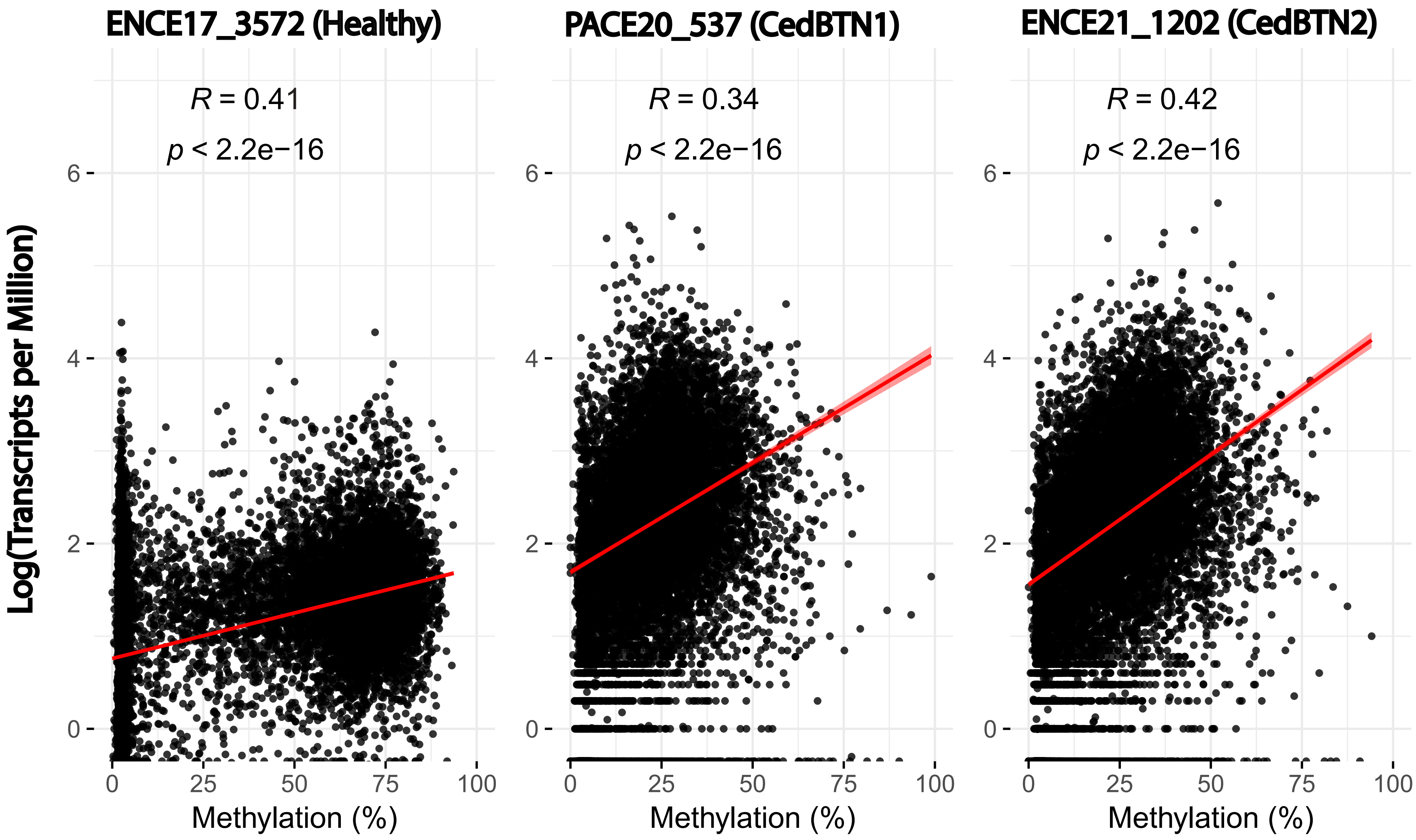
ENCE17_3572 (Healthy)
PACE20_537 (CedBTN1)
ENCE21_1202 (CedBTN2)
R = 0.41
p < 2.2e−16
R = 0.34
p < 2.2e−16
R = 0.42
p < 2.2e−16
Log(Transcripts per Million)
0
2
4
6
0
25
50
75
100
Methylation (%)

Supplementary Figure 2: Relationship between intragenic CpG methylation profile and expression levels. Scatter plot and trend line (Pearson's correlation) illustrating the association between intragenic CpG methylation (X-axis) and mRNA expression (Y-axis, depicted as log10(TPM)) in healthy (ENCE17_3572), CedBTN1 (PACE20_537), and CedBTN2 (ENCE21_1202) samples. The line depicts linear regression, and the shaded area denotes the +-95% confidence interval. Correlation coefficient values and adjustments are provided in each scatter plot.

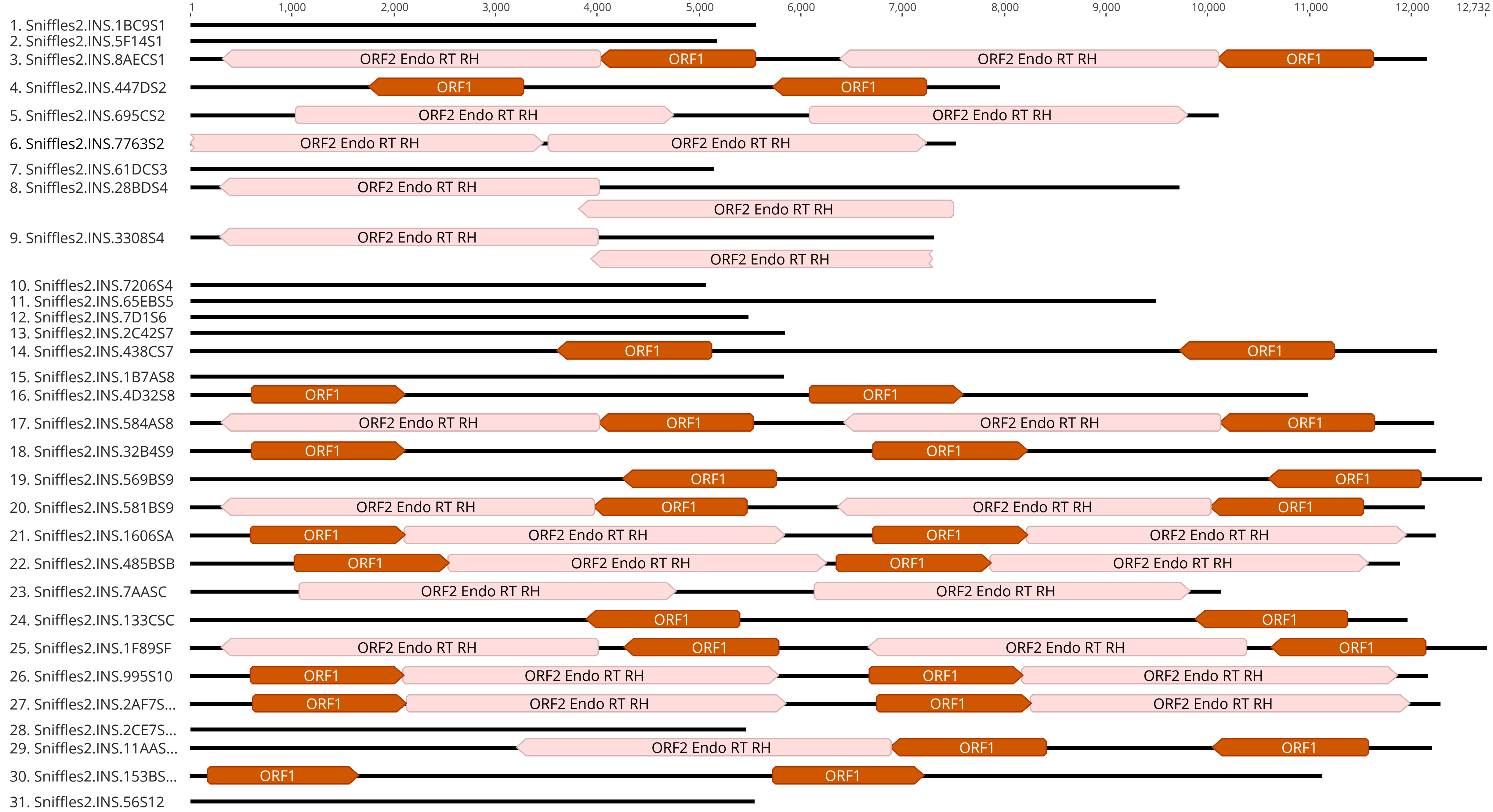

1
1,000
2,000
3,000
4,000
5,000
6,000
7,000
8,000
9,000
10,000
11,000
12,000
12,732
1. Sniffles2.INS.1BC9S1
2. Sniffles2.INS.5F14S1
3. Sniffles2.INS.8AECS1
ORF2 Endo RT RH
ORF1
ORF2 Endo RT RH
ORF1
4. Sniffles2.INS.447DS2
ORF1
ORF1
5. Sniffles2.INS.695CS2
ORF2 Endo RT RH
ORF2 Endo RT RH
6. Sniffles2.INS.7763S2
ORF2 Endo RT RH
ORF2 Endo RT RH
7. Sniffles2.INS.61DCS3
8. Sniffles2.INS.28BDS4
ORF2 Endo RT RH
ORF2 Endo RT RH
9. Sniffles2.INS.3308S4
ORF2 Endo RT RH
ORF2 Endo RT RH
10. Sniffles2.INS.7206S4
11. Sniffles2.INS.65EBS5
12. Sniffles2.INS.7D1S6
13. Sniffles2.INS.2C42S7
14. Sniffles2.INS.438CS7
ORF1
ORF1
15. Sniffles2.INS.1B7AS8
16. Sniffles2.INS.4D32S8
ORF1
ORF1
17. Sniffles2.INS.584AS8
ORF2 Endo RT RH
ORF1
ORF2 Endo RT RH
ORF1
18. Sniffles2.INS.32B4S9
ORF1
ORF1
19. Sniffles2.INS.569BS9
ORF1
ORF1
20. Sniffles2.INS.581BS9
ORF2 Endo RT RH
ORF1
ORF2 Endo RT RH
ORF1
21. Sniffles2.INS.1606SA
ORF1
ORF2 Endo RT RH
ORF1
ORF2 Endo RT RH
22. Sniffles2.INS.485BSB
ORF1
ORF2 Endo RT RH
ORF1
ORF2 Endo RT RH
23. Sniffles2.INS.7AASC
ORF2 Endo RT RH
ORF2 Endo RT RH
24. Sniffles2.INS.133CSC
ORF1
ORF1
25. Sniffles2.INS.1F89SF
ORF2 Endo RT RH
ORF1
ORF2 Endo RT RH
ORF1
26. Sniffles2.INS.995S10
ORF1
ORF2 Endo RT RH
ORF1
ORF2 Endo RT RH
27. Sniffles2.INS.2AF7S...
ORF1
ORF2 Endo RT RH
ORF1
ORF2 Endo RT RH
28. Sniffles2.INS.2CE7S...
29. Sniffles2.INS.11AAS...
ORF2 Endo RT RH
ORF1
ORF1
30. Sniffles2.INS.153BS...
ORF1
ORF1
31. Sniffles2.INS.56S12

Supplementary Figure 3: Detection of genomic insertions of the retrotransposon CedCL24 using nanopore long-reads and Sniffles2 in the genome of a representative CedBTN2 sample (ENCE21_1202H). Features such as open reading frames (ORF) and repeats were annotated based on a sequence identity higher than 80% using Geneious. The colour scheme for the annotations matches that of Figure 4 in the main manuscript.

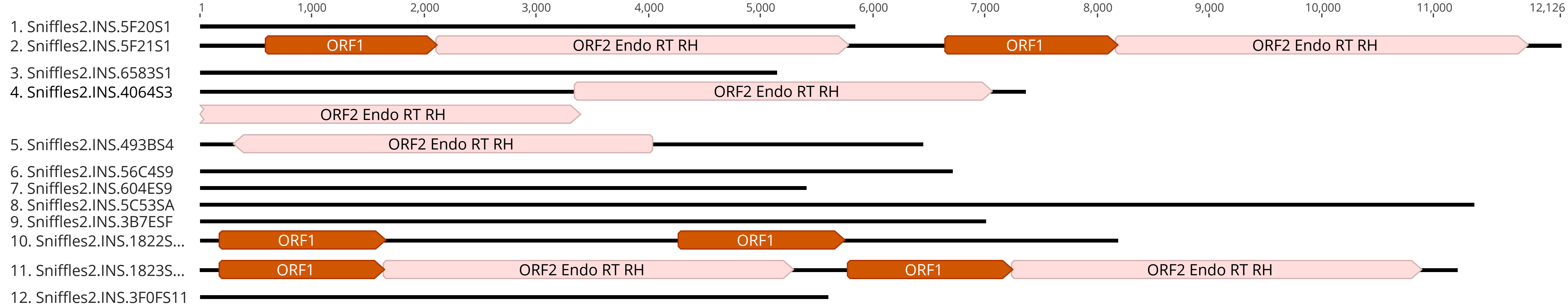
1
1,000
2,000
3,000
4,000
5,000
6,000
7,000
8,000
9,000
10,000
11,000
12,126
1. Sniffles2.INS.5F20S1
2. Sniffles2.INS.5F21S1
ORF1
ORF2 Endo RT RH
ORF1
ORF2 Endo RT RH
3. Sniffles2.INS.6583S1
4. Sniffles2.INS.4064S3
ORF2 Endo RT RH
ORF2 Endo RT RH
5. Sniffles2.INS.493BS4
ORF2 Endo RT RH
6. Sniffles2.INS.56C4S9
7. Sniffles2.INS.604ES9
8. Sniffles2.INS.5C53SA
9. Sniffles2.INS.3B7ESF
10. Sniffles2.INS.1822S...
ORF1
ORF1
11. Sniffles2.INS.1823S...
ORF1
ORF2 Endo RT RH
ORF1
ORF2 Endo RT RH
12. Sniffles2.INS.3F0FS11

Supplementary Figure 4: Detection of genomic insertions of the retrotransposon CedCL24 using nanopore long-reads and Sniffles2 in the genome of a representative CedBTN1 sample (PACE20_537H). Features such as open reading frames (ORF) and repeats were annotated based on a sequence identity higher than 80% using Geneious. The colour scheme for the annotations matches that of Figure 4 in the main manuscript.

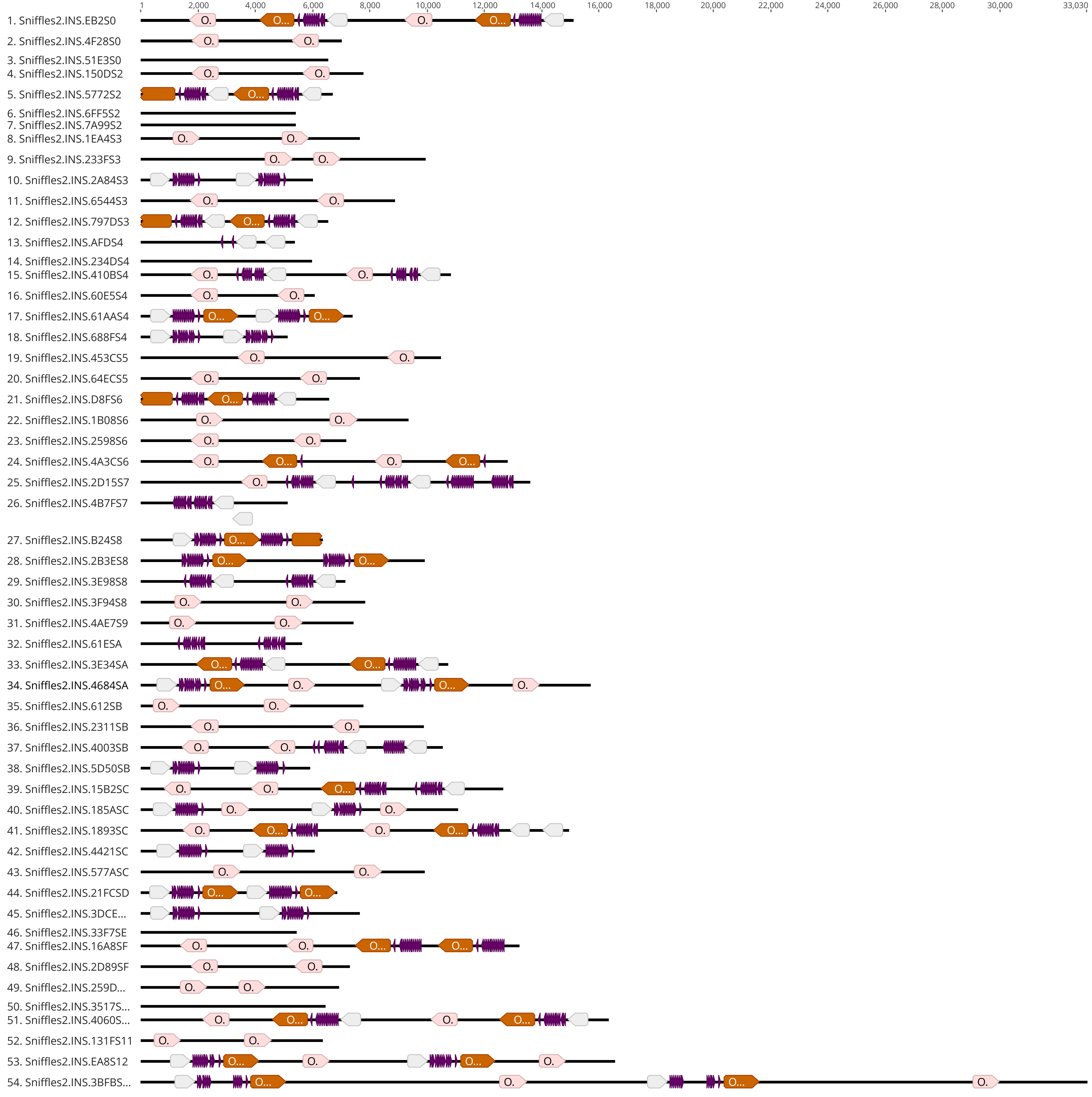
1
2,000
4,000
6,000
8,000
10,000
12,000
14,000
16,000
18,000
20,000
22,000
24,000
26,000
28,000
30,000
33,030
1. Sniffles2.INS.EB2S0
2. Sniffles2.INS.4F28S0
3. Sniffles2.INS.51E3S0
4. Sniffles2.INS.150DS2
5. Sniffles2.INS.5772S2
6. Sniffles2.INS.6FF5S2
7. Sniffles2.INS.7A99S2
8. Sniffles2.INS.1EA4S3
9. Sniffles2.INS.233FS3
10. Sniffles2.INS.2A84S3
11. Sniffles2.INS.6544S3
12. Sniffles2.INS.797DS3
13. Sniffles2.INS.AFDS4
14. Sniffles2.INS.234DS4
15. Sniffles2.INS.410BS4
16. Sniffles2.INS.60E5S4
17. Sniffles2.INS.61AAS4
18. Sniffles2.INS.688FS4
19. Sniffles2.INS.453CS5
20. Sniffles2.INS.64ECS5
21. Sniffles2.INS.D8FS6
22. Sniffles2.INS.1B08S6
23. Sniffles2.INS.2598S6
24. Sniffles2.INS.4A3CS6
25. Sniffles2.INS.2D15S7
26. Sniffles2.INS.4B7FS7
27. Sniffles2.INS.B24S8
28. Sniffles2.INS.2B3ES8
29. Sniffles2.INS.3E98S8
30. Sniffles2.INS.3F94S8
31. Sniffles2.INS.4AE7S9
32. Sniffles2.INS.61ESA
33. Sniffles2.INS.3E34SA
34. Sniffles2.INS.4684SA
35. Sniffles2.INS.612SB
36. Sniffles2.INS.2311SB
37. Sniffles2.INS.4003SB
38. Sniffles2.INS.5D50SB
39. Sniffles2.INS.15B2SC
40. Sniffles2.INS.185ASC
41. Sniffles2.INS.1893SC
42. Sniffles2.INS.4421SC
43. Sniffles2.INS.577ASC
44. Sniffles2.INS.21FCSD
45. Sniffles2.INS.3DCE...
46. Sniffles2.INS.33F7SE
47. Sniffles2.INS.16A8SF
48. Sniffles2.INS.2D89SF
49. Sniffles2.INS.259D...
50. Sniffles2.INS.3517S...
51. Sniffles2.INS.4060S...
52. Sniffles2.INS.131FS11
53. Sniffles2.INS.EA8S12
54. Sniffles2.INS.3BFBS...

Supplementary Figure 5: Detection of genomic insertions of the retrotransposon CedCL34 using nanopore long-reads and Sniffles2 in the genome of a representative CedBTN2 sample (ENCE21_1202H). Features such as open reading frames (ORF) and repeats were annotated based on a sequence identity higher than 80% using Geneious. The colour scheme for the annotations matches that of Figure 4 in the main manuscript.

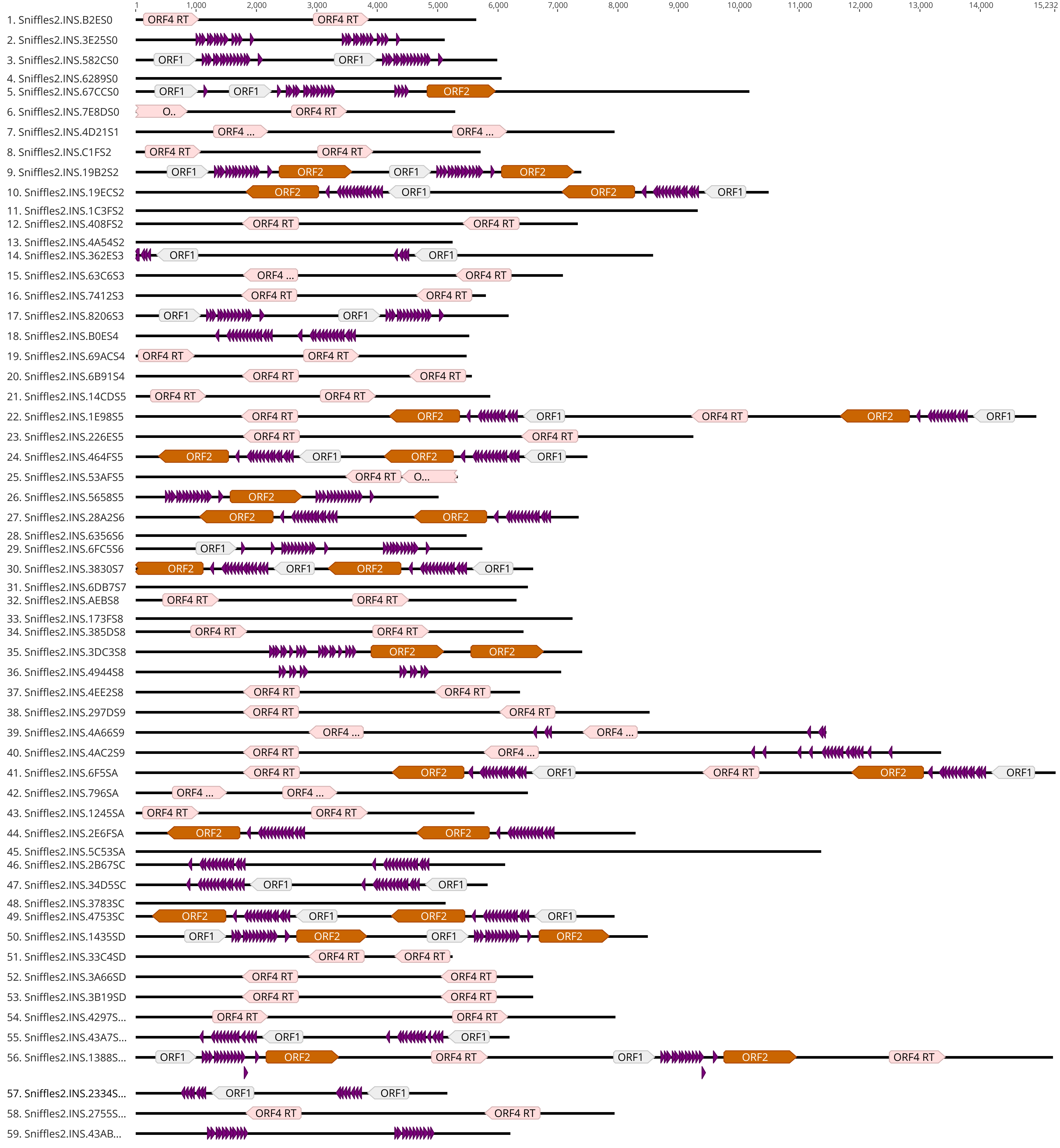

1
1,000
2,000
3,000
4,000
5,000
6,000
7,000
8,000
9,000
10,000
11,000
12,000
13,000
14,000
15,232
1. Sniffles2.INS.B2ES0 ORF4 RT ORF4 RT
2. Sniffles2.INS.3E25S0
3. Sniffles2.INS.582CS0 ORF1 ORF1
4. Sniffles2.INS.6289S0
5. Sniffles2.INS.67CCS0 ORF1 ORF1 ORF2
6. Sniffles2.INS.7E8DS0 O.. ORF4 RT
7. Sniffles2.INS.4D21S1 ORF4 ... ORF4 ...
8. Sniffles2.INS.C1FS2 ORF4 RT ORF4 RT
9. Sniffles2.INS.19B2S2 ORF1 ORF2 ORF1 ORF2
10. Sniffles2.INS.19ECS2 ORF2 ORF1 ORF2 ORF1
11. Sniffles2.INS.1C3FS2
12. Sniffles2.INS.408FS2 ORF4 RT ORF4 RT
13. Sniffles2.INS.4A54S2
14. Sniffles2.INS.362ES3 ORF1 ORF1
15. Sniffles2.INS.63C6S3 ORF4 ... ORF4 RT
16. Sniffles2.INS.7412S3 ORF4 RT ORF4 RT
17. Sniffles2.INS.8206S3 ORF1 ORF1
18. Sniffles2.INS.B0ES4
19. Sniffles2.INS.69ACS4 ORF4 RT ORF4 RT
20. Sniffles2.INS.6B91S4 ORF4 RT ORF4 RT
21. Sniffles2.INS.14CDS5 ORF4 RT ORF4 RT
22. Sniffles2.INS.1E98S5 ORF4 RT ORF2 ORF1 ORF4 RT ORF2 ORF1
23. Sniffles2.INS.226ES5 ORF4 RT ORF4 RT
24. Sniffles2.INS.464FS5 ORF2 ORF1 ORF2 ORF1
25. Sniffles2.INS.53AFS5 ORF4 RT O...
26. Sniffles2.INS.5658S5 ORF2
27. Sniffles2.INS.28A2S6 ORF2 ORF2
28. Sniffles2.INS.6356S6
29. Sniffles2.INS.6FC5S6 ORF1
30. Sniffles2.INS.3830S7 ORF2 ORF1 ORF2 ORF1
31. Sniffles2.INS.6DB7S7
32. Sniffles2.INS.AEBS8 ORF4 RT ORF4 RT
33. Sniffles2.INS.173FS8
34. Sniffles2.INS.385DS8 ORF4 RT ORF4 RT
35. Sniffles2.INS.3DC3S8 ORF2 ORF2
36. Sniffles2.INS.4944S8
37. Sniffles2.INS.4EE2S8 ORF4 RT ORF4 RT
38. Sniffles2.INS.297DS9 ORF4 RT ORF4 RT
39. Sniffles2.INS.4A66S9 ORF4 ... ORF4 ...
40. Sniffles2.INS.4AC2S9 ORF4 RT ORF4 ...
41. Sniffles2.INS.6F5SA ORF4 RT ORF2 ORF1 ORF4 RT ORF2 ORF1
42. Sniffles2.INS.796SA ORF4 ... ORF4 ...
43. Sniffles2.INS.1245SA ORF4 RT ORF4 RT
44. Sniffles2.INS.2E6FSA ORF2 ORF2
45. Sniffles2.INS.5C53SA
46. Sniffles2.INS.2B67SC
47. Sniffles2.INS.34D5SC ORF1 ORF1
48. Sniffles2.INS.3783SC
49. Sniffles2.INS.4753SC ORF2 ORF1 ORF2 ORF1
50. Sniffles2.INS.1435SD ORF1 ORF2 ORF1 ORF2
51. Sniffles2.INS.33C4SD ORF4 RT ORF4 RT
52. Sniffles2.INS.3A66SD ORF4 RT ORF4 RT
53. Sniffles2.INS.3B19SD ORF4 RT ORF4 RT
54. Sniffles2.INS.4297S... ORF4 RT ORF4 RT
55. Sniffles2.INS.43A7S... ORF1 ORF1
56. Sniffles2.INS.1388S... ORF1 ORF2 ORF4 RT ORF1 ORF2 ORF4 RT
57. Sniffles2.INS.2334S... ORF1 ORF1
58. Sniffles2.INS.2755S... ORF4 RT ORF4 RT
59. Sniffles2.INS.43AB...

Supplementary Figure 6: Detection of genomic insertions of the retrotransposon CedCL34 using nanopore long-reads and Sniffles2 in the genome of a representative CedBTN1 sample (PACE20_537H). Features such as open reading frames (ORF) and repeats were annotated based on a sequence identity higher than 80% using Geneious. The colour scheme for the annotations matches that of Figure 4 in the main manuscript.
